# Anatomical characteristics of mitral isthmus and its spatial relationship with the esophagus in patients undergoing atrial fibrillation ablation using CT angiography

**DOI:** 10.3389/fcvm.2025.1461744

**Published:** 2025-02-10

**Authors:** Yilin Pan, Hong Zeng, Xin Liu, Xiaohang Fu, Liyuan Pan, Yanjing Wang

**Affiliations:** ^1^ Department of Cardiology, China-Japan Union Hospital of Jilin University, Changchun, China; ^2^ Department of Critical Care Medicine, Beijing Anzhen Hospital, Capital Medical University, Beijing, China; ^3^ Department of Radiology, China-Japan Union Hospital of Jilin University, Changchun, China

**Keywords:** atrial fibrillation, ablation, mitral isthmus, esophageal injury, CT angiography

## Abstract

**Background:**

This study examines the anatomical characteristics of the mitral isthmus (MI) and its spatial relationship with the esophagus in patients undergoing atrial fibrillation ablation, using cardiovascular computed tomographic angiography (CTA). Understanding this relationship is crucial to minimize the risk of esophageal injuries during ablation procedures.

**Methods:**

The investigation included 300 participants, divided into 200 subjects in the experimental group undergoing atrial fibrillation ablation and 100 in the control group. Detailed CTA scans were used to assess the MI's structure and proximity to the esophagus, employing various measurements like the MI's endocardial length, depth, and its relation to adjacent esophageal anatomy.

**Results:**

The study revealed significant differences in the MI's length and distance measurements between the experimental and control groups, with the former showing greater dimensions, potentially influencing ablation strategies. A substantial proportion of patients exhibited close proximity or direct contact between the MI and the esophagus, emphasizing the importance of pre-procedural imaging in identifying risks for esophageal damage.

**Conclusions:**

Pre-procedural cardiovascular CTA provides essential insights into the MI's anatomical details and its relation to the esophagus, aiding in the customization of ablation strategies to enhance procedural safety and efficacy. The findings highlight the significance of tailored imaging assessments to mitigate esophageal injury risks in atrial fibrillation ablation.

## 1 Introduction

Atrial fibrillation (AF) is the most prevalent clinical arrhythmia (1). Catheter ablation has emerged as a standard and effective treatment strategy for AF, demonstrating marked superiority over antiarrhythmic drugs in improving clinical symptoms, and enhancing the overall quality of life (2–6).

For patients with persistent AF, the guidelines recommend performing pulmonary vein isolation and considering additional ablation targeting the sustaining substrate of AF (7, 8). The mitral isthmus (MI), located between the left inferior pulmonary vein (LIPV) and the mitral annulus, is a prevalent site for additional linear ablation in AF cases. Given the proximity of the MI region to crucial anatomical structures such as the esophagus, pulmonary arteries and airways, understanding its unique anatomical and morphological variations is imperative.

Atrio-esophageal fistula (AEF) is a rare but severe complication associated with AF radiofrequency ablation procedures. The incidence of AEF is reported to be between 0.1% and 0.04% per ablation procedure (9, 10). However, its manifestation is almost universally fatal, with mortality rates nearing 100% (11, 12). The pivotal element contributing to esophageal injury is the anatomical proximity between the esophagus and the left atrium (LA). Due to this intimate anatomical relationship, ablative energy may permeate the left atrial muscle, causing detrimental effects on the esophageal wall (10). When performing additional linear ablation of the MI—which initiates at the mitral annulus and terminates near the posterior wall of the LA, adjacent to the LIPV—the risk of esophageal injury is significantly increased due to the close spatial relationship between the MI and the esophagus.

Currently, left atrial computed tomographic angiography (CTA) is routinely used in preoperative assessments for patients scheduled for AF ablation. This study used CTA to examine the anatomical details of the MI and its spatial relationship with the adjacent esophagus in patients undergoing AF ablation. The aim was to enhance the understanding of the MI ablation line and its anatomical interrelation with the adjacent esophagus before AF ablation. This enhanced insight might assist operators in choosing appropriate ablation techniques and energy settings, ultimately aimed at minimizing the risk of postoperative esophageal injury complications.

## 2 Methods

### 2.1 Patient selection and criteria

This study encompassed 200 patients consecutively enrolled between July 2020 and December 2021 in the Cardiovascular Department of China-Japan Union Hospital of Jilin University. All participants in the experimental group (AF group) underwent radiofrequency ablation. The control group comprised 100 non-AF individuals who also underwent CTA.

The following are the exclusion criteria for the patients:

(1) Patients with esophageal tumors or other conditions that could lead to significant esophageal dilation or thickening.
(2) Patients with a dilated esophagus, defined as an esophageal diameter greater than 20 mm at any level on CTA. To avoid normal variations in the esophageal lumen, such as those caused by recent food or liquid intake, all patients were instructed to fast for at least 6 h before undergoing CTA.
(3) Patients with pulmonary vein anomalies, including common pulmonary veins or pulmonary veins with three or more branches, as identified by CTA.

The study was approved by the Ethics Committee of the China-Japan Union Hospital of Jilin University (approval number: 2023122704), and conducted in accordance with the Declaration of Helsinki. Confidentiality of patient data was strictly maintained throughout the research.

### 2.2 Data acquisition and analysis

The study employed a third-generation dual-source CT (SOMATOM Force, Siemens, Germany), incorporating both respiratory and electrocardiogram gating. A retrospective electrocardiogram-gated scanning approach was deployed, using iohexol (Omnipaque, 370; 100 ml, GE Healthcare Inc., USA) as the contrast medium. Patients were positioned supine and interfaced with a dual-barrel high-pressure injector. For scan localization, patients were aligned foot-first with arms elevated, holding their breath. The positioning line was established 1.0 cm above the tracheal bifurcation to the cardiac diaphragmatic surface. The high-pressure injector facilitated the intravenous administration of 70 ml of the contrast agent through the right cubital vein at a flow rate of 4.5 ml/s, followed by a 50-ml flush of normal saline at a consistent rate. The region of interest was determined at the LA's level, using an intelligent tracking system to initiate scanning upon detecting a CT attenuation shift to 100 HU.

The scanning parameters comprised a slice thickness of 0.625 mm, dual-energy scanning tube voltages of 90 and Sn150 kV, an effective current of 280 mAs, a tube rotation time of 0.33 s, and a pitch ranging from 0.16 to 0.2.

Data measurements were performed by an independent observer with over 10 years of training and experience in interpreting CTA images. To assess the reliability of the measurements, inter-observer and intra-observer reproducibility were evaluated as follows:For inter-observer reproducibility, a second experienced independent observer performed repeated measurements on the same 30 patients' CTA images, using the same methods and measurement standards. For intra-observer reproducibility, the same observer performed a second round of measurements on the same images to assess consistency over time. Multiple pivotal parameters were diligently assessed for meticulous quantification of the MI. The subsequent sections delineate the specialized methods employed for these precise measurements:

(1) easurement of MI endocardial length:

The exact distance of the MI endocardial length was quantified by measuring the distance (in millimeters) from the bottom of the LIPV to the MI.

(2) easurement of MI linear distance:

Employing a consistent plane as used in the initial measurement, the linear extension of the MI was ascertained (in millimeters).

(3) Measurement of MI depth:

The depth of the MI was determined by measuring the distance (in millimeters) from the specified point on the line from the second measurement to the profoundest endocardial point of the MI.

**Figure 1:**
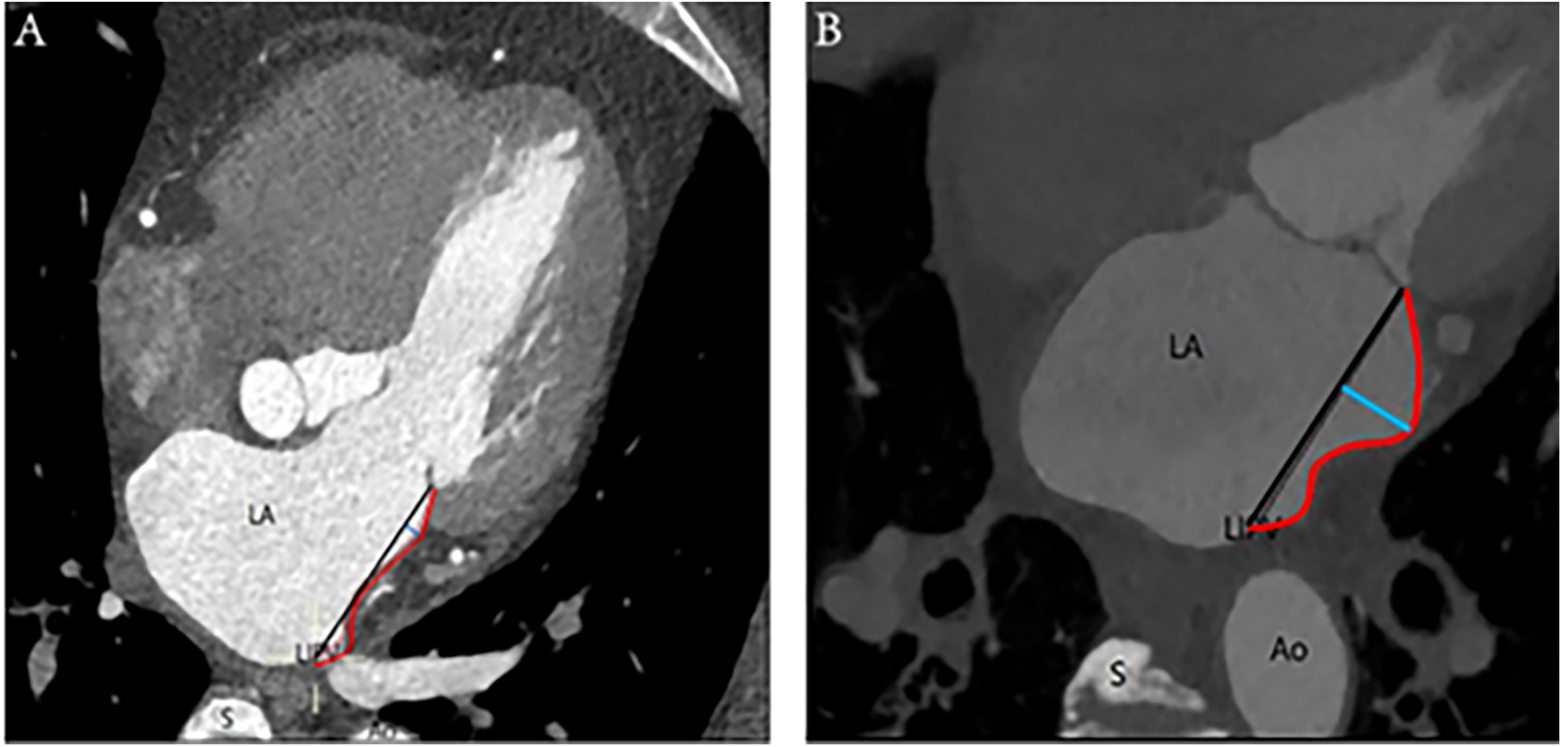
Linear and curved MI: **(A)** On the left is an illustration of a linear-shaped MI, whereas **(B)** on the right is a depiction of a curved-shaped MI. The annotations are as follows: Ao, aorta; LA, left atrium; LIPV, left inferior pulmonary vein; S, spine; red curve, distance of MI; black straight line, length of MI; blue straight line, depth of MI.

Depths ≤2 mm characterized the MI as linear, whereas depths >2 mm designated the MI as curved, based on clinical observation and anatomical experience (Figure 1).

**Figure 2:**
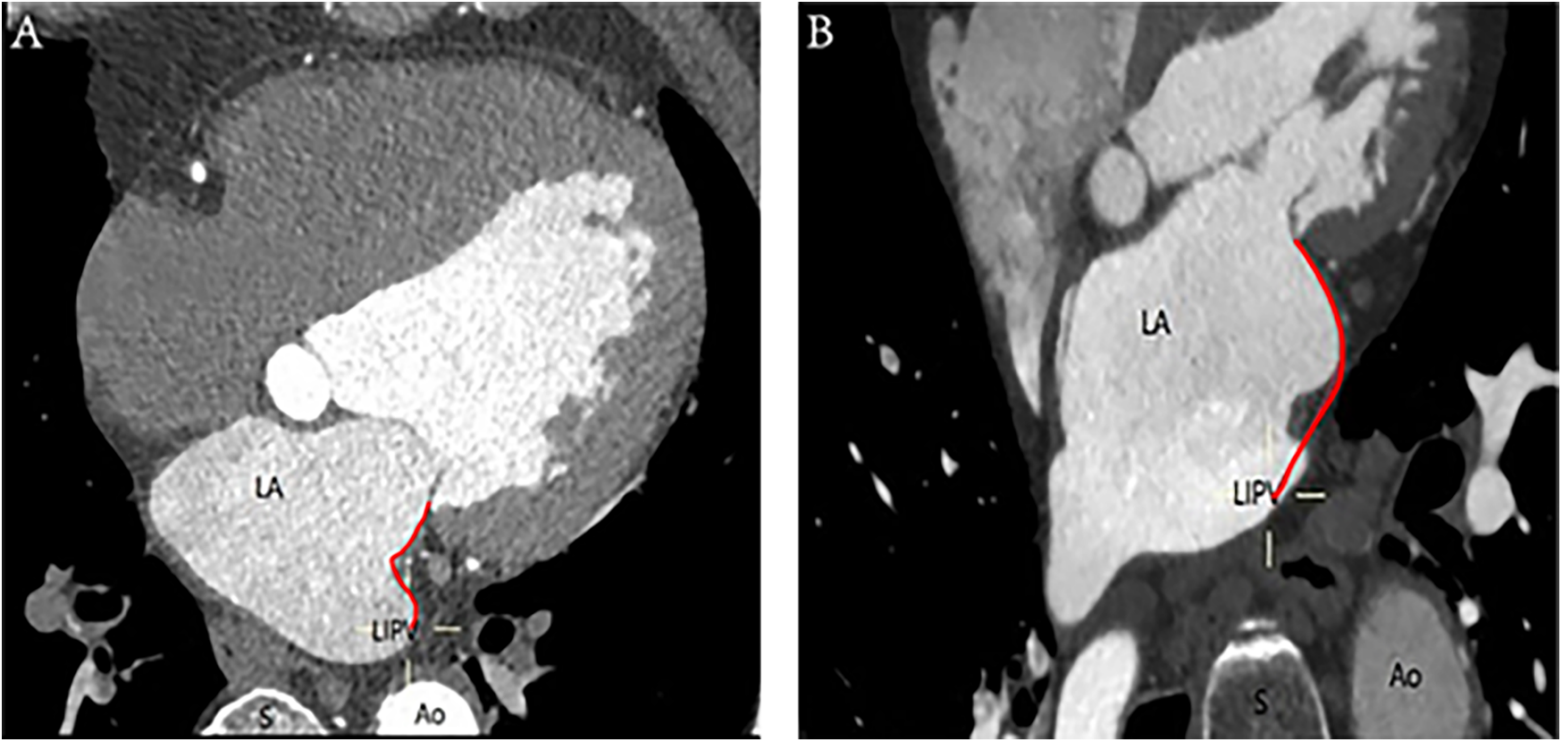
Ridge-shaped and pouch-shaped MI: **(A)** The left image depicts a ridge-shaped MI, whereas **(B)** the right image illustrates a pouch-shaped MI. The annotations are as follows: Ao, aorta; LA, left atrium; LIPV, left inferior pulmonary vein; S, spine; red curve, distance of MI.

A ridge-shaped MI is identified when the endocardial contour prominently extends along a vertical line, forming a slender band on the surface. Conversely, the pouch-shaped morphology comprises the vestibule and the pouch, enhancing the granularity of the anatomical characterization of the MI, as depicted in Figure 2.

**Figure 3:**
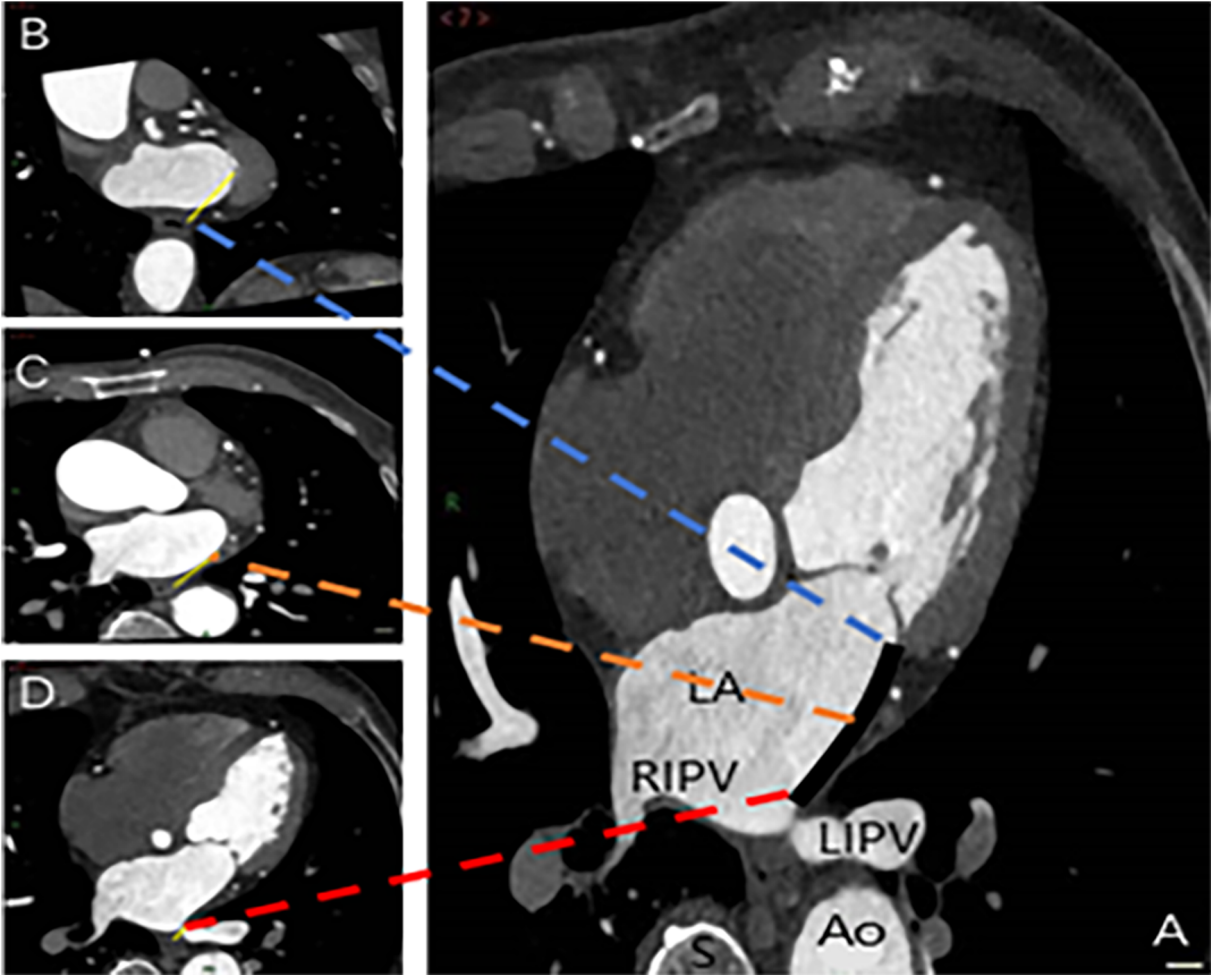
Spatial measurements of MI and its proximity to the esophagus. The measurements were taken at three key locations: Level 1 (intersection of the upper end of MI and LIPV) in image D, Level 2 (midpoint of MI) in image C, and Level 3 (level of the mitral annulus) in image B. The annotations are as follows: Ao, aorta; LA, left atrium; LIPV, left inferior pulmonary vein; RIPV, right inferior pulmonary vein; S, spine; black solid line, MI distance measurement; yellow solid line, distance from the three levels to the esophagus.

**Figure 4:**
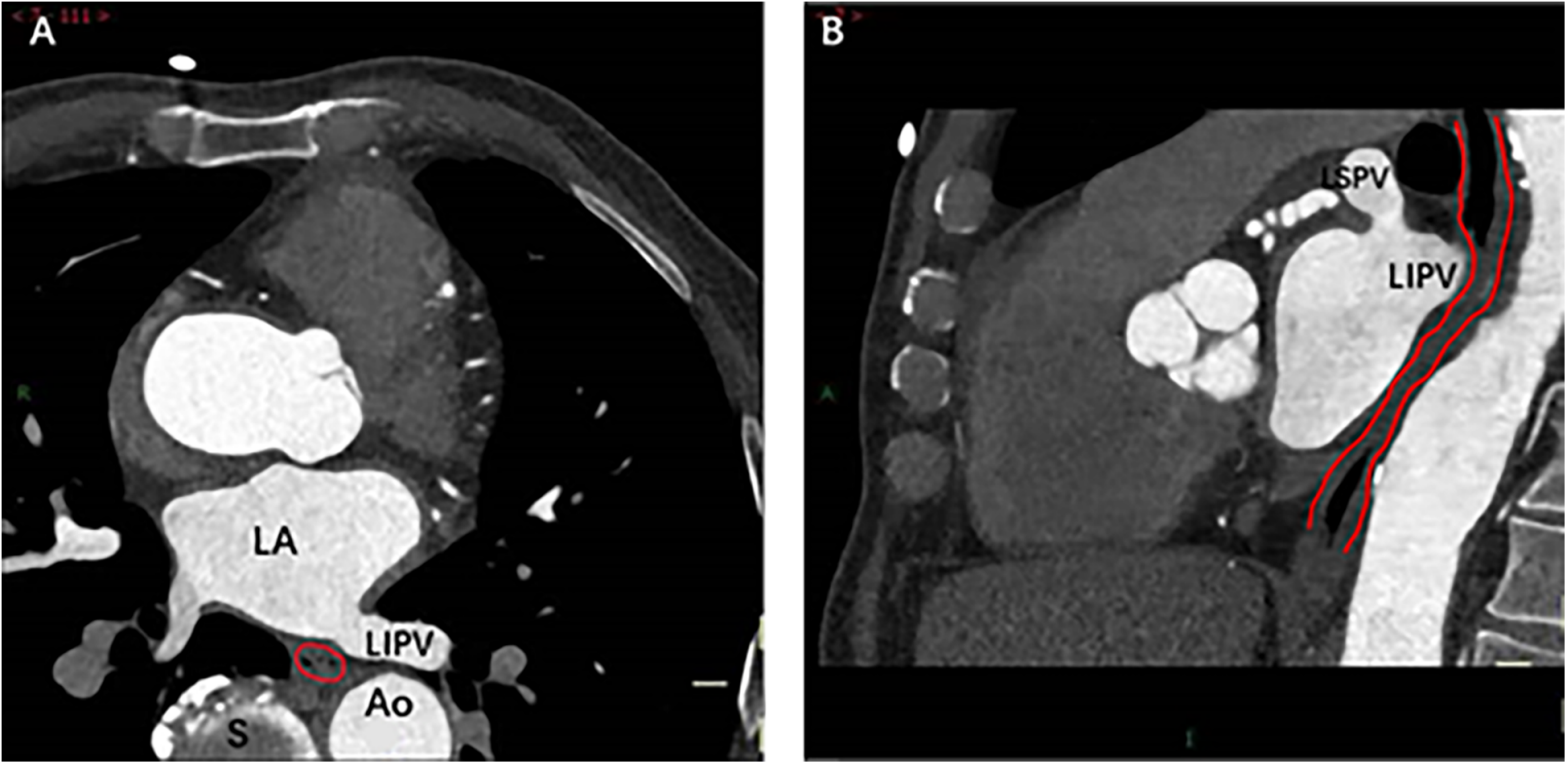
Spatial relationship between MI and esophagus. The cross-sectional (**A**) and sagittal (**B**) images show direct contact between the bottom of the left lower pulmonary vein and the esophagus (indicated by red lines). Right image: As the esophagus descends, the spatial distance between it and MI gradually increases. Ao, Aorta; LA, left atrium; LIPV, Left lower pulmonary vein; LSPV, Left superior pulmonary vein; S, spine.

The spatial measurements of the MI were meticulously captured using the post-processing techniques of multiplanar reformation and volume rendering facilitated by CT, aiming to assess its proximity to the surrounding esophagus. The measurements were systematically recorded at three strategic locations: the intersection of the upper end of the MI and the LIPV (Level 1), the midpoint of the MI (Level 2), and the level of the mitral annulus (Level 3) (Figure 3). Cross-sectional and sagittal images, as illustrated in Figure 4, were utilized to accurately determine the proximity of the MI to the esophagus at these levels, providing a detailed assessment of their spatial relationship.

The measurements were made to ascertain the minimal spatial distances between the esophagus and the LIPV. Based on these measurements, patients were stratified into two risk groups according to the proximity of the esophagus to the LIPV: a high-risk group (distance from the esophagus to the LIPV < 5 mm) and a low-risk group (distance from the esophagus to the LIPV ≥ 5 mm). To further analyze the impact of age, patients were also stratified into two subgroups based on age: age ≤65 years and age >65 years.

### 2.3 Statistical analysis

IBM SPSS Statistics 25.0 was utilized for statistical analyses. Continuous variables underwent normality assessment using the Kolmogorov–Smirnov test. Data adhering to normal distribution were presented as mean ± standard deviation (*x* ± *S*), with group comparisons via independent-sample or Welch's *t* tests. Non-normal continuous data were expressed as median[M (P_25_, P_75_)], analyzed using the Mann–Whitney U test. Categorical data, reported as frequencies or proportions, were evaluated using chi-square, corrected chi-square, or Fisher's exact tests. Pearson's coefficient analyzed correlations in normally distributed bivariate data, while Spearman's coefficient was applied to non-normal variables. A *p* value <0.05 was considered statistically significant. Both inter-observer and intra-observer reproducibility were evaluated using the intraclass correlation coefficient (ICC), with ICC values ≥0.75 indicating good agreement between the observers.

## 3 Results

### 3.1 Patient characteristics

The study included 300 participants divided into 2 groups: 200 in the AF group and 100 in the control group. A comparison between the AF and control groups revealed no statistically significant differences in demographic and clinical characteristics such as age, sex distribution, history of smoking, alcohol consumption, hypertension, diabetes, and coronary artery disease (*p* > 0.05 for all variables). Likewise, laboratory parameters such as triglycerides (TG), total cholesterol (TC), and low-density lipoprotein cholesterol (LDL-C) exhibited no statistically significant differences between the two groups (*p* > 0.05 for all parameters) (Table 1).

**Table 1:** Comparison of general information between two groups of patients.

	AF group	Control group	*P*
*n*	200	100	–
Age (year)	62.63 ± 10.22	61.20 ± 14.24	0.370
Sex (men, %)	125 (62.5)	52 (52.0)	0.081
Smoking (cases, %)	60 (30.0)	32 (32.0)	0.723
Alcohol consumption (cases, %)	33 (16.5)	11 (11.0)	0.204
Hypertension (cases, %)	86 (43.0)	54 (54.0)	0.072
Diabetes (cases, %)	32 (16.0)	16 (16.0)	>0.999
Coronary heart disease (cases, %)	92 (46.0)	57 (57.0)	0.072
TG (mmol/L)	1.31 (0.97–1.78)	1.36 (1.06–2.44)	0.097
TC (mmol/L)	4.42 (3.62–5.16)	4.40 (3.75–5.26)	0.659
LDL-C (mmol/L)	2.56 (1.95–3.28)	2.69 (2.09–3.28)	0.345

### 3.2 Reproducibility testing

The ICC analysis revealed that the intra-observer ICC values for the MI-related anatomical measurements and the three esophageal distance measurements ranged from 0.943 to 0.995, while the inter-observer ICC values ranged from 0.937 to 0.981 (Table 2).

**Table 2:** Reproducibility of MI anatomical measurements and esophageal distances.

Measurement	Intra-Observer ICC	Inter-Observer ICC
MI Endocardial length	0.976	0.975
MI Linear length	0.973	0.981
MI Depth	0.992	0.978
Level 1	0.995	0.947
Level 2	0.982	0.980
Level 3	0.943	0.937

### 3.3 Imaging results

#### 3.3.1 Comparison of Mi structure and morphology between the two groups

Both the length and distance of the MI were significantly greater in the AF group than in the control group (length: 42.17 ± 9.52 mm vs. 36.45 ± 8.28 mm, *p* < 0.001; distance: 39.20 ± 8.40 mm vs. 34.06 ± 7.35 mm, *p* < 0.001). However, the depths of the MI in both groups did not exhibit a significant difference [3.91 (1.95–6.20) mm in the AF group and 4.25 (2.57–7.14) mm in the control group, *p* = 0.105].

In terms of MI morphology, 27% of participants in the AF group exhibited linear-type MIs and 73% showed curvilinear-type MIs. In comparison, these proportions were 22% and 78%, respectively, in the control group. The distribution of ridge- and pouch-type MIs also did not differ significantly between the two groups, with *p* > 0.05 in all comparisons (Table 3).

**Table 3:** Comparison of MI structure and morphology between the two groups.

	AF group	Control group	*P*
MI length (mm)	42.17 ± 9.52	36.45 ± 8.28	<0.001
MI distance (mm)	39.20 ± 8.40	34.06 ± 7.35	<0.001
MI depth (mm)	3.91 (1.95,6.20)	4.25 (2.57,7.14)	0.105
Linear-type MI (cases, %)	50 (25.0)	22 (22.0)	0.566
Curved-type MI (cases, %)	150 (75.0)	78 (78.0)	
Ridge-shaped MI (cases, %)	44 (22.0)	18 (18.0)	0.420
Pouch-shaped MI (cases, %)	156 (78.0)	82 (82.0)	

#### 3.3.2 Comparison of MI and esophagus spatial distance based on LIPV distance and age

In this study, patients were divided into high-risk and low-risk groups based on the proximity of the esophagus to the LIPV. Each group was further stratified by age (≤65 years and >65 years).

In the high-risk group, patients aged ≤65 years had a significantly greater minimal spatial distance between the MI and the esophagus at Level 1 in the AF group compared to the control group [AF group: 3.82 (2.46, 4.31) mm vs. control group: 2.67 (1.82, 3.70) mm, *p* = 0.018]. However, no significant differences were observed at Level 2 and Level 3 [Level 2: 21.15 (18.38, 24.84) mm vs. 19.49 (17.55, 23.24) mm, *p* = 0.277; Level 3: 34.20 ± 6.31 mm vs. 31.99 ± 5.76 mm, *p* = 0.132]. In patients aged >65 years, no significant differences were found between the AF group and control group at any of the levels [Level 1: AF group: 3.10 ± 1.02 mm vs. control group: 3.29 ± 1.09 mm, *p* = 0.555; Level 2: 22.13 ± 6.60 mm vs. 23.02 ± 6.10 mm, *p* = 0.610; Level 3: 34.87 ± 8.07 mm vs. 35.65 ± 5.52 mm, *p* = 0.682].

In the low-risk group, patients aged ≤65 years showed no significant differences in the minimal spatial distance between the MI and the esophagus at Level 1 [AF group: 10.36 (6.71, 14.43) mm vs. control group: 7.61 (5.96, 11.95) mm, *p* = 0.103], Level 2 [AF group: 26.1 (22.33, 31.10) mm vs. control group: 24.78 (21.44, 31.22) mm, *p* = 0.559], or Level 3 [AF group: 37.92 ± 8.44 mm vs. control group: 37.16 ±7.95 mm, *p* = 0.658]. For patients aged >65 years, the AF group had a smaller minimal spatial distance at Level 1 compared to the control group, but this difference was not statistically significant [AF group: 7.92 (5.79, 13.23) mm vs. control group: 10.74 (7.67, 13.71) mm, *p* = 0.225]. No significant differences were found at Level 2 [AF group: 27.43 ± 7.89 mm vs. control group: 28.58 ± 7.79 mm, *p* = 0.578] or Level 3 [AF group: 40.29 ± 9.84 mm vs. control group: 38.21 ± 6.59 mm, *p* = 0.311] (Table 4).

**Table 4:** Comparison of spatial distance between esophagus and LIPV across different age groups and measurement levels in AF and control patients.

Group	Age	Measurement level	AF group	Control group	*P*
High-risk	≤65	Level 1	3.82 (2.46,4.31)	2.67 (1.82,3.70)	0.018
		Level 2	21.15 (18.38,24.84)	19.49 (17.55,23.24)	0.277
		Level 3	34.20 ± 6.31	31.99 ± 5.76	0.132
	>65	Level 1	3.10 ± 1.02	3.29 ± 1.09	0.555
		Level 2	22.13 ± 6.60	23.02 ± 6.10	0.610
		Level 3	34.87 ± 8.07	35.65 ± 5.52	0.682
Low-risk	≤65	Level 1	10.36 (6.71,14.43)	7.61 (5.96,11.95)	0.103
		Level 2	26.1 (22.33,31.10)	24.78 (21.44,31.22)	0.559
		Level 3	37.92 ± 8.44	37.16 ± 7.95	0.658
	>65	Level 1	7.92 (5.79,13.23)	10.74 (7.67,13.71)	0.225
		Level 2	27.43 ± 7.89	28.58 ± 7.79	0.578
		Level 3	40.29 ± 9.84	38.21 ± 6.59	0.311

## 4 Discussion

This study aimed to explore the anatomical characteristics of the MI and its spatial relationship with the esophagus in patients undergoing AF ablation using cardiovascular CTA. The main findings of this study were as follows: (1) Patients with AF exhibited a significantly longer MI length and greater MI distance compared to controls, which could increase procedural complexity during ablation; (2) A notable proportion of patients had a minimal spatial distance of less than 5 mm between the MI and the esophagus, particularly in the high-risk group, emphasizing the importance of pre-procedural imaging; (3) Younger patients (≤65 years) in the high-risk group demonstrated a significantly greater distance at Level 1 compared to controls, possibly influenced by epicardial adipose tissue (EAT). These findings are further elaborated and discussed below.

### 4.1 Analysis of the characteristics of MI structure and morphology

This study assessed the endocardial length of the MI in two distinct groups: the AF and control groups, yielding a mean length of 42.17 ± 9.52 mm and 36.45 ± 8.28 mm, respectively. The linear distance of the MI was determined to be 39.20 ± 8.40 mm in the AF group and 34.06 ± 7.35 mm in the control group, aligning closely with the findings of Wittkampf et al. (13), who reported an analogous MI length of 35 ± 7 mm in a cadaveric anatomical study. In the present study, the AF group demonstrated an enhanced MI length and linear distance compared with the control group. Complementary observations of Scherr et al. (14) suggested that the MI length acted as a determinant independent risk factor, obstructing the attainment of a definitive bidirectional conduction block amid ablation procedures. This indicated that patients with AF possessing extended MI segments might experience protracted ablation periods and an escalation in procedural intricacies, necessitating thoughtful strategic planning and execution.

A significant finding of this study based on the measurements of MI depth was as follows: a modest proportion of MIs was linear type, irrespective of the presence of AF, whereas a substantial majority (>75%) exhibited curvilinear configurations. The prevalence of curvilinear MIs was due to their non-flat endocardial surfaces, complicating the attainment of optimal catheter–target contact during ablation. Furthermore, most patients (>78%) displayed a pouch-like morphology, contrasting with a ridge-like structure. Pouch-like configurations, forming concave atrial vestibules and recesses, posed potential hindrances to effective catheter–tissue contact during ablation, escalating the risk of catheter entrapment. Yokokawa et al. (15) confirmed these findings, illustrating increased challenges in achieving a complete bidirectional block when the pouch-like MI depth exceeded 10 mm. These observations underscored the critical influence of the distinct anatomical features of the MI on procedural complexity and success.

### 4.2 Analysis of the spatial relationship between the MI and the esophagus

In a detailed comparative analysis of patients in the high-risk group, those aged <65 years exhibited a significantly greater minimal spatial distance between the upper border of the MI and the LIPV junction (Level 1) compared to the control group. This finding suggests that for younger patients in the high-risk group, the proximity of the esophagus to the LIPV may be notably altered in patients with AF, potentially contributing to a greater distance at Level 1. A conceivable explanation for the extended distance at Level 1 in patients with AF is the presence of EAT. Previous studies, including those by Le Jemtel et al. (16)and Ahn et al. (17), have highlighted EAT volume as a significant intrinsic risk factor for AF, with pronounced left atrial (LA) and EAT volumes identified in patients with AF.

The accumulation of EAT in AF patients may create additional mechanical separation between the MI and the esophagus, resulting in a greater distance at Level 1 compared to those without AF. This phenomenon is less pronounced at Level 2 and Level 3, where the inherent distances between the MI and the esophagus are greater, making it less likely for EAT to cause a notable effect. Although this hypothesis provides a plausible explanation for the observed findings, further research is necessary to explore the complex relationship between AF and EAT. More comprehensive studies could help clarify whether EAT directly contributes to the altered spatial relationships observed in the high-risk AF patients, particularly those under the age of 65.

In some patients, the MI measurement points near the upper border of the LIPV were found to be less than 5 mm from the esophagus, placing these tissues at high risk of thermal injury during ablation. Given this close anatomical proximity, careful consideration of tailored ablation strategies is essential to minimize complications and enhance procedural safety.

Several practical approaches can be employed to enhance procedural safety in these cases. Modifying ablation parameters is essential, as studies suggest that reducing ablation power (<25–30 W) and shortening lesion duration (<20 s) can significantly lower the risk of esophageal injury (18, 19). Alternatively, high-power short-duration protocols (e.g., 40–50 W for 5–15 s) produce more controlled and superficial lesions, reducing thermal penetration (19). Recent advancements in esophageal protection techniques also highlight the potential of thermal control devices. For instance, the IMPACT study demonstrated that using the ensoETM thermal control device during ablation procedures significantly reduced the incidence of esophageal thermal injuries without affecting procedural efficacy or duration (20). This finding underscores the importance of incorporating esophageal protection measures, such as active cooling, into procedural planning to minimize complications and improve patient outcomes. Alternative energy modalities, such as cryoablation and pulsed-field ablation (PFA), also show promise in reducing esophageal complications (21, 22). PFA, in particular, uses non-thermal mechanisms to induce tissue necrosis and has been shown to be safer for the esophagus in both preclinical and early clinical studies (23). Additionally, intraoperative esophageal temperature monitoring with probes provides real-time feedback to adjust energy delivery when necessary, while esophageal cooling systems can help maintain the esophageal wall temperature below the injury threshold (24, 25). Despite these benefits, challenges such as probe positioning or potential mechanical injury must also be considered (26).

Integrating these strategies with individualized planning and patient selection can minimize esophageal injury risks and improve outcomes in high-risk patients. This study translates anatomical findings into actionable guidance for interventional cardiologists managing complex ablation cases.

## 5 Limitations

This study had certain limitations. First, the retrospective design of the study may limit the generalizability of the results and the applicability of the findings to clinical practice. Second, due to the lack of detailed intraoperative data, such as ablation power and duration, and patient outcome data, the clinical implications of these findings, particularly in terms of risk stratification or the practical application of CTA-based measurements before pulmonary vein ablation, remain unclear. Future research will integrate these data to better understand the relationship between mitral isthmus characteristics and ablation difficulty and assess whether these findings can contribute to improving procedural safety and efficacy. Third, potential confounders in the CTA-based measurements, such as variations in esophageal distension, vertebral changes, and anatomical differences, could have introduced biases. Finally, CTA has limitations in accurately measuring very thin structures like the myocardial thickness of the mitral isthmus, and we plan to explore this further using Cardiovascular Magnetic Resonance in future studies.

## 6 Conclusions

Cardiovascular CTA has equipped interventional physicians with a detailed understanding of the anatomical characteristics of MI ablation lines in patients with AF and their spatial relationship with the adjacent esophagus, before beginning ablation procedures. This invaluable insight has facilitated the meticulous selection of personalized ablation protocols and energy parameters, consequently reducing the potential risks and complications associated with esophageal injuries.

## Data Availability

The original contributions presented in the study are included in the article/Supplementary Material, further inquiries can be directed to the corresponding authors.
